# Validation of Kannada Version of the Amsterdam Preoperative Anxiety and Information Scale (APAIS)

**DOI:** 10.7759/cureus.63486

**Published:** 2024-06-29

**Authors:** Madhu Srinivasaiah, Nandini Mysore Gopalswamy, Harini Krishna, Venkatesh Murthy Kallur Thimmarayappa

**Affiliations:** 1 Anesthesiology, Dr. Chandramma Dayananda Sagar Institute of Medical Education and Research, Kanakapura, IND

**Keywords:** apais, kannada, validity, translation, preoperative anxiety

## Abstract

Background and aim: Preoperative anxiety is a complex and subjective phenomenon, most commonly observed in patients posted for elective surgery. The Amsterdam Preoperative Anxiety Information Scale (APAIS) has been widely used in preoperative settings to measure anxiety quickly. This study aimed to translate the APAIS into Kannada and to evaluate its psychometric properties.

Method: The study involved forward and backward translation of APAIS into Kannada followed by a cognitive interview of 10 patients by clinical psychologist to confirm the correct translation. The translated version was then administered to 240 patients who were posted for elective surgery along with the Visual Analogue Scale for Anxiety (VAS-A). Reliability and internal consistency were assessed by calculating Cronbach’s alpha. Construct validity was assessed by principal component analysis and confirmatory factor analysis. Criteria validity was assessed by evaluating the correlation between APAIS and VAS-A.

Result: The Kannada-translated version of APAIS was completed by 224 patients posted for elective surgery. Cronbach’s alpha was 0.82 and 0.83 for anxiety and need for information items, respectively. Root mean square error of approximation (RMSEA), comparative fit index (CFI), and Tucker-Lewis index (TLI) were 0.07, 0.98, and 0.96, respectively, for three-factor model, indicating it to be best fit for Kannada version of APAIS.

Conclusion: The Kannada version of APAIS is a valid and reliable tool for measuring preoperative anxiety in patients undergoing elective surgery.

## Introduction

Anxiety is one of the most commonly observed psychological manifestations of the patients posted for surgery. Preoperative anxiety is observed in about 11-80% of surgical patients [1,2]. Preoperative anxiety can lead to negative consequences like difficult venous access, increased requirement of intraoperative anesthetics, exaggerated postoperative pain, postoperative nausea and vomiting, prolonged hospital stay, and associated morbidity [3-5].

Preoperative anxiety is a subjective phenomenon and is influenced by various factors, which makes it difficult to assess objectively [6,7]. Several instruments are being used for measuring preoperative anxiety like the Spielberger State-Trait Anxiety Inventory (STAI), Hospital Anxiety and Depression (HAD) scale, Visual Analogue Scale for Anxiety (VAS-A), etc. In clinical psychology and psychiatry, STAI is used most frequently [8]. It is also used more often for assessing preoperative anxiety and is considered as gold standard [9]. However, in a clinical setting, especially in preoperative framework, the use of STAI is associated with an increase in time consumption. Another disadvantage is that the inventory is copyright-restricted and has to be purchased for regular use.

The Amsterdam Preoperative Anxiety and Information Scale (APAIS) is a freely available self-report questionnaire developed to assess preoperative anxiety. APAIS comprises six items, two items on anxiety about anesthesia, two on anxiety about surgery, and two related to the need for information. Each item is addressed by patients on a five-point Likert scale, with 1 being "not at all worrying" to 5 as "extremely worrying." APAIS was first developed in Dutch and has been validated in surgical patients [10]. The anxiety score of APAIS had shown a high correlation with STAI-state (0.74) in Dutch patients posted for surgery. The APAIS has been subsequently translated and validated in English, Japanese, German, French, Italian, Spanish, Chinese, and Portuguese [11-18].

The prevalence of preoperative anxiety in India is unknown; however, few studies indicate it to be around 30-50% [6,19]. In majority of these studies, no validated inventory was used for measuring preoperative anxiety. Kannada, an official language of Karnataka state of India, is used by around 64 million people across the world. The purpose of this study is to translate the APAIS into Kannada and to evaluate its psychometric properties.

## Materials and methods

The study was initiated after approval from the institutional ethics committee and was registered in the clinical trial registry of India. The study was conducted in accordance with the principles laid down in the Declaration of Helsinki. Patients were enrolled in the study after obtaining written informed consent in Kannada language. Patients posted for emergency surgeries, cesarean section, patients with a history of substance dependence/abuse, patients on anti-psychotropic medication, and patients with known psychiatric disorders were excluded. Patients posted for elective surgery with age >18 years and able to read and understand Kannada were included in the study.

The APAIS tool has six items, if we consider respondent to item ratio of 30:1, a sample size of 180 would be sufficient for the validation study. However in order to account for missed data and to have a fair sample, a sample size of 240 was considered for our study [20].

Patients posted for surgery underwent routine pre-anesthetic check-ups in OPD as per routine practice. During pre-anesthetic check-ups, patients deemed fit for surgery were accepted for surgery and those patients requiring optimization of comorbid conditions were advised accordingly. During this visit, patients were given necessary information regarding anesthesia as per routine practice by a concerned anesthesiologist. A list of patients deemed fit for surgery was generated a day prior to surgery.

On the day prior to surgery, the anesthesiologist made a visit to the respective ward, eligible subjects were explained about the aim of the study, and written informed consent was obtained. During the consultation with anesthesiologist, socio-demographic data and other clinical data that assess patients' fitness for surgery were collected. Patients were then provided the APAIS inventory followed by VAS-A. In VAS-A, patients were asked to express their anxiety on a scale of 0-100 mm with 0 indicating no anxiety and 100 indicating severe anxiety [21].

The scale

The APAIS is a self-administered questionnaire comprising six questions, two questions on anxiety related to anesthesia, two on anxiety related to surgery, and two on the need for information. The answers are recorded on a five-point Likert scale from 1 (not at all worrying) to 5 (extremely worrying). The scores of the anxiety subscales range from four to 20 and the need for information ranges from two to 10. A higher score is associated with more anxiety. In the study by Moerman et al, the cutoff for anxiety was a total anxiety scale score of eleven [10].

Validation process

The process of validation is comprised of two steps. The first step was to develop a Kannada version of APAIS that is semantically equivalent to the original version of APAIS. The second step involved the evaluation of psychometric properties of the Kannada version.

After obtaining authorization for the translation of APAIS to Kannada from the authors of original scale, forward translation was done by a bilingual senior anesthesiologist. This was reviewed by an expert followed by back translation by an independent translator. After the finalization of translated version, the instrument was administered to 10 surgical adult patients who underwent cognitive interview by a senior clinical psychologist to obtain an equivalent translated scale in Kannada.

To check if the Kannada version would maintain similar factor structure as in previous studies and to evaluate internal validity, confirmatory factor analysis was done. Bartlett’s test of sphericity and Kaiser-Meyer-Olkin measure of sampling adequacy were done to check if factor analysis could be performed on the data. Two-factor model as described in the original APAIS and the three-factor model as described in French APAIS validation were evaluated [10,14]. The root mean square error of approximation (RMSEA), comparative fit index (CFI), and Tucker-Lewis index (TLI) were used to check the adequacy of model. A CFI and TLI >0.9 and RMSEA <0.08 indicate a satisfactory fit of the data. Cronbach’s alpha was calculated to check for reliability and internal consistency.

Non-parametric Spearman’s correlation was used to determine the correlation between APAIS scores and VAS on anxiety. Mean scale scores were compared by student t-test. A p-value of <0.05 was considered significant. Statistical analysis was done by using Jamovi computer software version 2.3 (Sydney, Australia: The Jamovi Project 2024) and IBM SPSS statistics version 20.0 (Armonk, NY: IBM Corp.) for Windows.

## Results

Process of translation

The Kannada version of APAIS did not show any mismatches when back translated to English by a different bilingual health professional (table in the appendix). Cognitive interviews with 10 participants did not require rephrasing of the translated versions.

Characteristics of study subjects

The study included 240 subjects posted for elective surgery, of which 16 forms were incomplete and hence excluded from the final analysis. The characteristics of subjects are presented in Table 1. Subjects were categorized based on education status into Category A - primary education (20.98%), Category B - high school (33.03%), and Category C - above high school (45.98%).

**Table 1:** Characteristics of the patients. ASA: American Society of Anesthesiologists

Variables		Category A (primary)	Category B (high school)	Category C (above high school)
Gender	Male	18 (8%)	20 (8.9%)	43 (19.2%)
	Female	29 (12.9%)	54 (24.10%)	60 (26.8%)
ASA	I	30 (13.4%)	54 (24.1%)	81 (36.2%)
	II	17 (7.6%)	20 (8.9%)	22 (9.8%)
Comorbid illness	Present	17 (7.6%)	20 (8.9%)	22 (9.8%)
	Absent	30 (13.4%)	54 (24.1%)	81 (36.2%)
Previous surgery	Yes	27 (12%)	25 (11.2%)	37 (16.5%)
	No	20 (8.9%)	49 (21.9%)	66 (29.5%)
General surgery		25 (11.2%)	33 (14.7%)	48 (21.4%)
Obstetrics and gynecology		11 (4.9%)	28 (12.5%)	32 (14.3%)
Orthopedics		8 (3.6%)	9 (4%)	18 (8%)
Otorhinolaryngology		3 (1.3%)	4 (1.8%)	5 (2.2%)

Reliability and validity of APAIS Kannada version

Tests of Reliability and Internal Consistency

The internal consistency reliability was high. The Cronbach’s alpha for all items was 0.82 for anxiety-related items (AQ1, AQ2, AQ4, and AQ6), and for desire for information was 0.83.

Construct Validity

Bartlett’s test of sphericity (chi-square=0.559 and p<0.001) and Kaiser-Meyer-Olkin (KMO) measure of sampling adequacy 0.77 indicated that the data sample is adequate for factor analysis. A principal component analysis with varimax rotation revealed two factors, which accounted for 72.19% of variance. The communalities, factor loadings, and eigenvalue are shown in Table 2. Overall, inter-item correlations were good for APAIS items with weakest correlation between item six and item one.

**Table 2:** Factor structure, eigenvalue, and percent of variance in a two-factor model.

Factors	Items	Factor loading	Eigen value	Variance	Total variance
Factor 1	I am worried about anesthesia	0.79	3.311	55.19%	72.19%
	The anesthesia is constantly on my mind	0.86			
	I am worried about the procedure	0.85			
	The procedure is constantly on my mind	0.84			
Factor 2	I would like to know as much as possible about the anesthesia	0.88	1.02	16.99%	
	I would like to know as much as possible about the procedure	0.90			

Test for Dimensionality

Confirmatory factor analysis was done by evaluating two-factor model (as described in the original version) and three-factor model (as described in the French APAIS validation). The RMSEA, CFI, and TLI of both models are given in Table 3. The three-factor model was the best fit for the data.

**Table 3:** Confirmatory factor analysis. CFI: comparative fit index; RMSEA: root mean square error of approximation; TLI: Tucker-Lewis index

Models and items	Two-factor model	Three-factor model
Chi-square p-value	<0.001	0.04
CFI	0.95	0.98
TLI	0.92	0.96
RMSEA	0.11	0.07

Tests of Concurrent or External Validity

The APAIS global anxiety score was compared with VAS-A. The mean anxiety score significantly correlated with VAS-A with r=0.504 and p=0.001.

Anxiety and Need for Information

The anxiety comprised four questions, two questions on anxiety about anesthesia and two questions on anxiety about surgery. As three-factor model was the best fit for our data, we compared means of anxiety about anesthesia, anxiety about surgery, and need for information based on gender or comorbid condition or previous history of surgery. There was no significant difference across groups (Table 4).

**Table 4:** Comparison of means of anxiety and need for information in a three-factor model based on gender, history of comorbidity, and previous surgery.

Variables	Anxiety about anesthesia	p-Value	Anxiety about surgery	p-Value	Need for information	p-Value
Gender (n)						
Male (81)	2.96±1.04	0.15	2.88±1.51	0.09	3.80±1.97	0.69
Female (143)	3.17±1.07		3.80±1.68		3.91±1.95	
Comorbidity (n)						
Present (59)	3.14±0.94	0.71	3.36±1.84	0.19	4.03±2.19	0.46
Absent (165)	3.08±1.05		3.03±1.54		3.81±1.86	
History of previous surgery (n)						
Yes (88)	3.11±1.01	0.82	3.14±1.69	0.86	3.90±2.07	0.82
No (135)	3.08±1.03		3.10±1.59		3.84±1.87	

## Discussion

This study aimed to translate and validate APAIS for use in the Kannada-speaking population. The forward translation and backward translation were done by two independent individuals who were expert researchers proficient in both languages and cultures. Similar to the original study, principal component analysis with varimax rotation revealed two factors. The factor loadings were >0.79, indicating that items one, two, four, and five were related to anxiety, and items three and six were related to need for information.

The Kannada version of APAIS showed high reliability with Cronbach’s alpha >0.80 for anxiety and need for information scale similar to the original and other validation studies [10-18]. In contrast to validation in Chinese, German, Turkish, and original studies, confirmatory factor analysis suggested a three-dimensional model as the best fit for the Kannada version [10,13,17,22]. Our study results were similar to the French translation (CFI of 1.0) and Portuguese translation (CFI: 0.970 and TLI: 0.926). The RMSEA is an index that is responsive to sample size and complexity and a value of <0.05 is considered good and between 0.05 and 0.08 is considered acceptable [23,24]. In our study, a value of 0.07 indicated a fair fit of three-dimensional model as compared to two-dimensional model. This is similar to findings in French and Portuguese validation studies [14,18]. To have a more holistic view of the goodness of fit, it is recommended to use multiple fit indices which would account for sample size, model complexity, and various other considerations in a particular study [24].

The hypothesis that females are more anxious than males, or patients with a previous history of surgery would be less anxious was evaluated in our study and showed no significant difference. This is in contrast to findings in the original study and might be due to the skewed distribution of groups, cultural differences, or paternalistic type of doctor-patient relationship.

One of the limitations of our study is that we did not compare APAIS to other anxiety tools as we do not have a valid tool to measure preoperative anxiety in Kannada and also there is enough evidence supporting APAIS as a preoperative anxiety tool. However, we compared it with VAS-A which showed a moderate level of correlation. Also, the variability observed in our study could be due to the inclusion of patients posted for major and minor surgeries, posted under general anesthesia or regional anesthesia. Further studies considering these factors will probably give more insight into the level of anxiety prior to surgery.

## Conclusions

The results of our study indicate that the Kannada version of the APAIS is valid and reliable. This translated version of APAIS with a three-dimensional structure can be valuable in measuring preoperative anxiety in patients undergoing elective surgeries. In this era of digitalization and readily available information, it has become a very important tool in assessing preoperative anxiety. The Kannada version of APAIS which is a simple, brief, and quick screening inventory, can further be used to estimate the prevalence of preoperative anxiety. Thus, this helps in planning appropriate interventions to alleviate anxiety, which finally would improve patient satisfaction and outcome of surgery.

## Appendices

**Table 5:** List of six APAIS items. APAIS: Amsterdam Preoperative Anxiety Information Scale

S. no.	Items (English)	Items (Kannada)
1	I am worried about the anesthetic	ಅರಿವಳಿಕೆಯ ಬಗ್ಗೆ ನನಗೆ ತುಂಬಾ ಚಿಂತೆ ಇದೆ
2	The anesthetic is on my mind continually	ಅರಿವಳಿಕೆಯು ಸದಾ ನನ್ನನು ಕಾಡುತ್ತದೆ
3	I would like to know as much as possible about the anesthetic	ಅರಿವಳಿಕೆಯ ಬಗ್ಗೆ ಸಾಧ್ಯವಾದಷ್ಟು ಹೆಚ್ಚು ತಿಳಿಯ ಬಯಸುತ್ತೇನೆ
4	I am worried about the procedure	ಶಸ್ತ್ರಚಿಕಿತ್ಸೆಯ ಬಗ್ಗೆ ನನಗೆ ತುಂಬಾ ಚಿಂತೆ ಇದೆ
5	The procedure is on my mind continually	ಶಸ್ತ್ರಚಿಕಿತ್ಸೆಯು ಸದಾ ನನ್ನನು ಕಾಡುತ್ತದೆ
6	I would like to know as much as possible about the procedure	ಶಸ್ತ್ರಚಿಕಿತ್ಸೆಯ ಬಗ್ಗೆ ಸಾಧ್ಯವಾದಷ್ಟು ಹೆಚ್ಚು ತಿಳಿಯ ಬಯಸುತ್ತೇನೆ
